# Terrestrial contributions to Afrotropical aquatic food webs: The Congo River case

**DOI:** 10.1002/ece3.5594

**Published:** 2019-08-27

**Authors:** David X. Soto, Eva Decru, Jos Snoeks, Erik Verheyen, Lora Van de Walle, Jolien Bamps, Taylor Mambo, Steven Bouillon

**Affiliations:** ^1^ Department of Earth and Environmental Sciences KU Leuven Leuven Belgium; ^2^ Section Vertebrates, Ichthyology Royal Museum for Central Africa Tervuren Belgium; ^3^ Laboratory of Biodiversity and Evolutionary Genomics KU Leuven Leuven Belgium; ^4^ OD Taxonomy and Phylogeny Royal Belgian Institute of Natural Sciences Brussels Belgium; ^5^ Department Biology, Evolutionary Ecology University of Antwerp Antwerpen Belgium; ^6^ Centre de Surveillance de la Biodiversité Université de Kisangani Kisangani Democratic Republic of Congo

**Keywords:** allochthony, fish communities, invertebrates, stable isotopes, stomach contents, terrestrial inputs, tropical rivers

## Abstract

1. Understanding the degree to which aquatic and terrestrial primary production fuel tropical aquatic food webs remains poorly understood, and quantifying the relative contributions of autochthonous and allochthonous inputs is methodologically challenging. Carbon and nitrogen stable isotope ratios (*δ* ^13^C, *δ* ^15^N) can provide valuable insights about contributions of terrestrial resources and trophic position, respectively, but this approach has caveats when applied in typical complex natural food webs.
2. Here, we used a combination of C, N, and H (*δ* ^2^H) stable isotope measurements and Bayesian mixing models to estimate the contribution of terrestrial (allochthonous) and aquatic (autochthonous) inputs to fish and invertebrate communities in the Congo River (and some tributaries).
3. Overall, our results show that we gained power to distinguish sources by using a multiple tracer approach and we were able to discriminate aquatic versus terrestrial sources (esp. including hydrogen isotopes). Fish *δ* ^2^H values were clearly correlated with their food preferences and revealed a high level of variation in the degree of allochthony in these tropical aquatic communities.
4. At the community level, it is clear that terrestrial C_3_ plants are an important source fueling the Congo River food web. However, in order to better constrain source contribution in these complex environments will require more robust constraints on stable isotope values of algal and methane‐derived C sources.

## Graphical abstract

Our study points toward a general significance of terrestrial C_3_ plants (allochthonous sources) for the fish and invertebrate communities in Afrotropical systems such as the Congo River basin. However, results showed a high variability of allochthony within communities of this large tropical aquatic system.

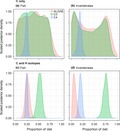

## 1 INTRODUCTION

Large tropical river food webs support a diverse and abundant fish assemblage with numerous and contrasting food preferences (Forsberg, Araujo‐Lima, Martinelli, Victoria, & Bonassi, 1993; Lundberg, Lewis, Saunders, & Mago‐leccia, 1987). Overall, in large rivers worldwide, algal organic matter can mainly support their food webs, except in systems with high suspended matter load (Roach, 2013). The main tropical rivers in the world, such as the Amazon, Orinoco, and Congo rivers, export high amounts of organic C inputs into the ocean annually with a minor expected contribution of autochthonous production (Coynel, Seyler, Etcheber, Meybeck, & Orange, 2005). Many tropical riverine fish species ingest large quantities of detritus (Bowen, 1983), and detritivorous fish and associated food webs are generally thought to rely on terrestrial energy inputs. However, a growing body of evidence has demonstrated that the importance of algae and other sources for the food chain, either directly or indirectly, is more important than traditionally thought, even in systems with large inputs of terrestrial matter (Brett et al., 2017). For instance, in the Orinoco River floodplain—an ecosystem dominated by vascular plants—Hamilton, Lewis, and Sippel (1992) suggested that algal production is a key energy source sustaining a diverse aquatic food web and Araujo‐Lima, Forsberg, Victoria, and Martinelli (1986) showed that detritivorous characiform fishes in the Amazon River feed on phytoplankton‐based food sources. Recent evidence has also shown that a large fraction of CH_4_ produced in freshwaters are typically oxidized by methane‐oxidizing bacteria before emission and these CH_4_‐derived carbon inputs can partially support both pelagic and benthic food webs in tropical (and temperate) aquatic ecosystems from invertebrates to fish species (Bastviken, Ejlertsson, Sundh, & Tranvik, 2003; Deines, Bodelier, & Eller, 2007; Sanseverino, Bastviken, Sundh, Pickova, & Enrich‐Prast, 2012).

There is a high variation of abiotic conditions that affects food webs in tropical freshwater systems, ranging from nutrient‐rich, white‐water rivers to nutrient‐poor, black‐water rivers with low pH, where high methane production can occur. Temporal changes in hydrological seasonality (wet vs. dry) and rhythmicity have also proven to be important mechanisms that control the trophic niche traits distribution and richness of tropical fish species (Fitzgerald, Winemiller, Sabaj Pérez, & Sousa, 2017; Jardine et al., 2015), likely due to seasonal fluctuations in food availability. On this respect, seasonal changes in allochthonous food availability (fruits, seeds) closely matched changes in dietary preferences of frugivorous fishes in the Amazon (Correa & Winemiller, 2014).

The answer to the question to which degree aquatic (autochthonous) and terrestrial (allochthonous) primary production fuel aquatic food webs is of fundamental importance when describing the ecological functioning of large aquatic ecosystems (Caraco, Bauer, Cole, Petsch, & Raymond, 2010; Cummins, 1974). While the importance of terrestrial production has been demonstrated in several lakes and rivers, there is no validated conceptual understanding of the factors that determine to which extent aquatic faunal communities rely on terrestrial and in situ aquatic production, particularly in tropical rivers. In addition, quantifying the relative contributions of these resources to food webs is methodologically challenging, since many of the frequently used approaches have limited resolving power. Tropical aquatic organisms can potentially ingest a large amount of detritus but the fraction of this source that is assimilated and incorporated into animal tissue cannot be inferred from conventional stomach content analysis. That is why carbon and nitrogen stable isotope ratios (*δ* ^13^C and *δ* ^15^N) are widely used to assess trophic linkages for aquatic food webs (reviewed in Boecklen, Yarnes, Cook, & James, 2011; Peterson & Fry, 1987) and to gain valuable insights regarding the relative contributions of allochthonous and CH_4_‐derived resources (Carpenter et al., 2005; Grey, 2016; Grey, Jones, & Sleep, 2001). Previous isotope studies in Afrotropical estuaries have shown a relative dependence of their food webs on the type of local vegetation cover, from C_3_‐ to C_4_‐dominated ecosystems (Abrantes, Barnett, & Bouillon, 2014; Abrantes, Barnett, Marwick, & Bouillon, 2013).

Stable hydrogen isotope ratios (*δ* ^2^H) have recently gained attention to complement “traditional” isotopes for aquatic food webs due to their potentially greater power to separate sources (Cole et al., 2011; Jardine, Kidd, & Cunjak, 2009; Newsome, Wolf, Bradley, & Fogel, 2017; Soto, Wassenaar, & Hobson, 2013). Their utility in the study of terrestrial versus aquatic sources contributions in aquatic food webs is based on the fact that *δ* ^2^H values are generally much lower in aquatic producers compared to terrestrial inputs such as leaf litter (Doucett, Marks, Blinn, Caron, & Hungate, 2007). Using the same basis, *δ* ^2^H values have also been used to examine the role of aquatic production to land ecosystems, therefore allowing to assess water‐to‐land energy transfer (Voigt, Lehmann, & Greif, 2015). In contrast, *δ* ^13^C and *δ* ^15^N values can show substantial overlap between terrestrial and aquatic sources, limiting their ability to disentangle their respective contributions (e.g., in the Orinoco and Amazon rivers, Araujo‐Lima et al., 1986; Hamilton et al., 1992). On the other hand, carbon isotope data are important tracers to distinguish C_3_ and C_4_ plant sources (Abrantes et al., 2014; Cerling et al., 1997) and methanotrophic inputs (Grey, 2016; Whiticar, 1999). Overall, new advances in analytical techniques such as stable hydrogen isotope analysis (Soto, Koehler, Wassenaar, & Hobson, 2017) and compound‐specific stable isotope approaches (e.g., amino acids, Liew et al., 2019) can potentially solve the common inability of separating aquatic and terrestrial sources.

The Congo River is the second longest river in Africa, as well as the world's second largest river that harbors the richest riverine fish species diversity in Africa. However, the Congo River basin remains a largely understudied ecosystem in spite of the enormous ecological value of their resources. A large fraction of the Congo basin is covered by dense forests, and the basin hosts a significant portion of the remaining African rainforest. A few recent intensive fish surveys have increased our knowledge of the ichthyofauna in the Congo River basin (e.g., Decru et al., 2017; Sonet et al., 2019; Stiassny, Brummett, Harrison, Monsembula, & Mamonekene, 2011; Van Steenberge, Vreven, & Snoeks, 2014), with 1,269 species (846 endemic) known to date (Winemiller et al., 2016). Moreover, fish represents the most important animal protein source to the riparian human population in this area. In spite of an increasing knowledge on the fish diversity, the main primary sources that fuel the food web are unknown. Our study aimed to evaluate to which extent the food web in the Congo River depends on autochthonous (aquatic, algal) and allochthonous (terrestrial plants) primary production, by combining the measurement of C, N, and H stable isotope ratios and gut content analysis information.

## 2 MATERIALS AND METHODS

### 2.1 Sample collection

**Figure 1:**
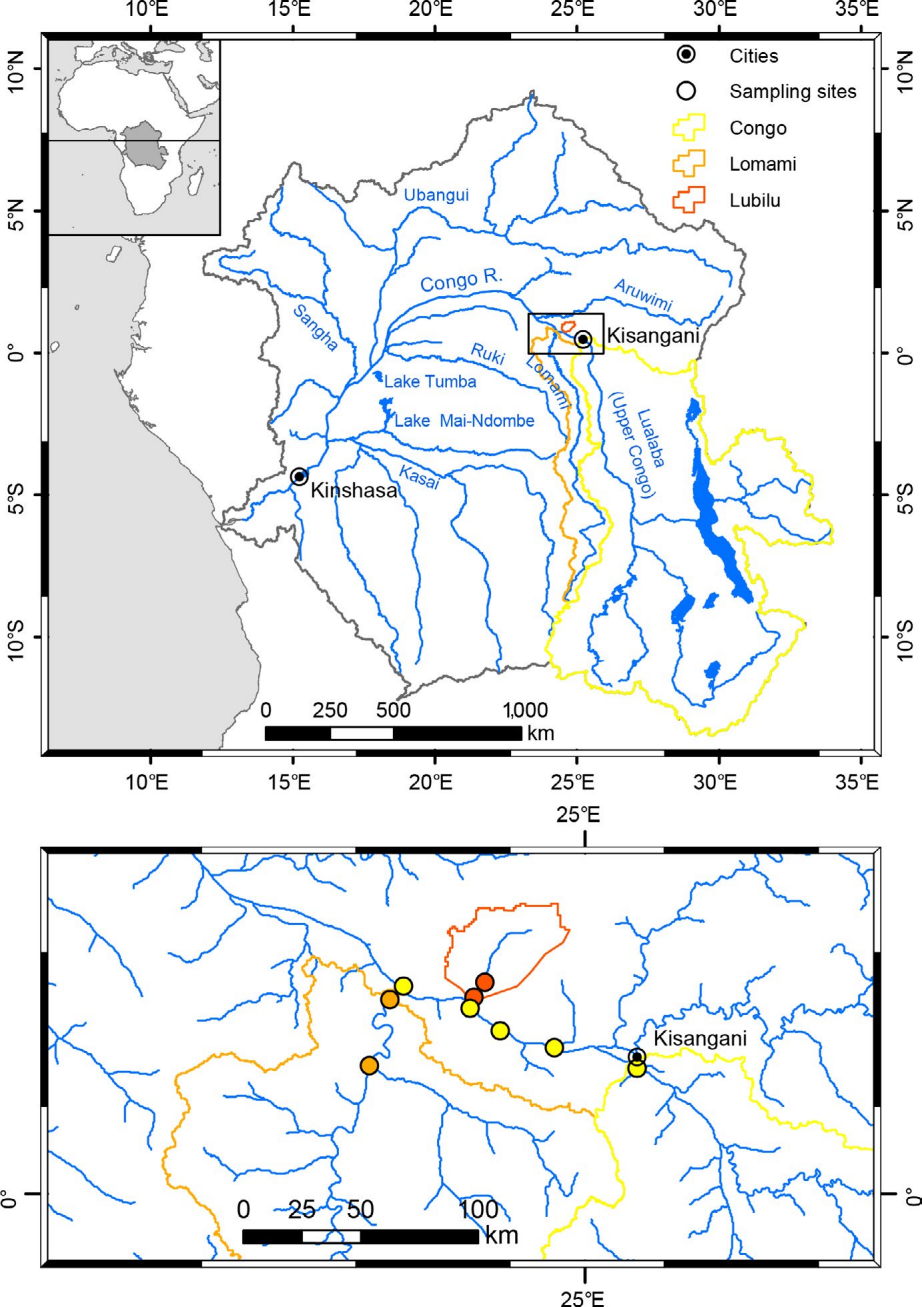
Map of the Congo River basin and the sampled subcatchments of the Congo River (Lubilu and Lomami) and mainstream Congo River (top). Sites within the Congo River basin sampled during the different campaigns (bottom)

Sampling of potential organic matter sources and consumers to investigate the reliance on terrestrial and aquatic resources was conducted in the central Congo River basin in a region downstream of Kisangani, close to the Yangambi Biosphere Reserve (Figure 1). We collected primary sources such as terrestrial litter from C_4_ grasses and C_3_ plants, riverine particulate organic matter (POM), and aquatic macrophytes. For POM collection, we filtered river water through a precombusted glass fiber filter (GFF), which was air‐dried in the field. For the consumers, we sampled aquatic and terrestrial invertebrates (whole body) and main fish species (dorsal muscle tissue). For each fish specimen, the standard length was measured. Samples were collected in two tributaries of the Congo River (Lubilu and Lomami) and in the mainstream Congo River, during three fieldtrips between 2012 and 2014 (November–December 2012, dry season; September 2013, wet season; March 2014, wet season), to account for seasonally contrasting hydrological conditions (Figure 1). One additional site (Lobaye, tributary of the Lomami) with low primary production and phytoplankton abundance was sampled in the field campaigns of 2012 and 2013. Environmental conditions (Secchi depth, pH, O_2_ saturation levels, Chl*a*) during sampling were (a) Lubilu: 1.3 ± 0.5 m, 4.8 ± 0.4, 53 ± 20%, 0.03 ± 0.004 µg/L, respectively; (b) Lomami: 0.7 ± 0.1 m, 5.9 ± 0.7, 78 ± 16%, 0.42 ± 0.10 µg/L, respectively; (c) mainstream Congo River: 0.5 ± 0.2 m, 6.9 ± 0.2, 82 ± 19%, 1.03 ± 0.43 µg/L, respectively; and (d) Lobaye: 0.9 ± 0.2 m, 5.1 ± 0.6, 87 ± 32%, 0.24 ± 0.07 µg/L, respectively. The river widths are in the order of 1–1.5 km for the mainstream Congo River, 400–500 m for the Lomami River, and 10–15 m for the Lubilu. In order to explore the potential of H isotope measurements, we selected the samples from the mainstream Congo River in 2012 because (a) we have an extensive set of water isotope data from the mainstream Congo River covering several years of regular measurements, (b) the natural C and N isotope variability in the food web components is higher and no significant differences in food web structure were found for the fish and invertebrate communities (see Results), and (c) by doing so, we avoid any temporal bias that could result from including samples from different years. The *δ* ^2^H values of the Congo River water (S. Bouillon and D.X. Soto, unpublished data, partially available through the GNIR data portal of the IAEA website) were measured as 0.0 ± 4.2‰ (average and *SD*, *n* = 103) from regular monitoring on the Congo River at Kisangani from December 2012 to May 2017 and −1.6 ± 1.6‰ (*n* = 58) from two transects across the mainstream Congo River from Kisangani to Kinshasa in December 2013 and June 2014 (see Borges et al., 2019, excluding downstream the confluence with the Kasai River).

### 2.2 Fish identification

All fishes were identified to the species level using the most recent available information. When identification keys were lacking, information of the species descriptions was used. In some difficult cases, specimens were compared with type specimens when available. In addition, DNA barcoding (COI, mtDNA) was used to facilitate and speed up the identification process. In total, 620 samples were successfully sequenced, using the protocol described in Decru et al. (2016). These sequences were compared to a reference database (Decru et al., 2016) from the same and neighboring regions, which allowed species delimitation and identification.

### 2.3 Gut content analysis

We examined gut contents for 247 fish specimens belonging to 86 species collected in March 2014 (wet season). Stomach content analysis represents a snapshot of what the fish has eaten in the recent past. The digestive tract was removed through a vertical section, and stomach and intestines were measured separately. Stomach contents were retrieved through a longitudinal section and by rinsing the stomach with 96% ethanol. When no discernible stomach content was present, the first 1/3th part of the intestines was examined.

Fish species were categorized in four feeding groups (herbivorous, omnivorous, invertebrate‐feeders, and piscivorous) and trophic levels (primary, secondary, and tertiary consumer) using stomach content and literature data (Table S1). Combining these two sources of information, we diminish the probability of any mismatch due to seasonally different feeding habits. Species, of which stomach contents were examined, were classified mostly based on the main detected food items. For the other species, this information was retrieved from literature (Table S1) or extrapolated on existing information for congeneric species. Omnivorous species were generally classified as secondary consumer, except when their diet also included fish. Stomach content items were identified and divided into different prey categories. For each stomach, contents were conserved for each prey category in 96% ethanol. All prey items were then oven‐dried (70°C for 24 hr) to obtain the dry weight of each prey category.

### 2.4 Stable isotope analysis

Samples were homogenized and were weighed along with in‐house standards in tin capsules for *δ* ^13^C and *δ* ^15^N, and in silver open capsules for *δ* ^2^H. In the case of POM samples, filters were acidified in a desiccator with concentrated HCl before analyses. Prior to *δ* ^2^H assays, all samples were treated with a 2:1 chloroform:methanol solution to remove bias associated with ^2^H‐depleted lipids (Sessions, Burgoyne, Schimmelmann, & Hayes, 1999; Soto et al., 2013). No solvent cleaning was conducted prior to *δ* ^13^C assays because the sample C/N ratios were close to those of bulk protein (Logan et al., 2008).

For *δ* ^13^C and *δ* ^15^N analyses, samples were flash combusted in a Thermo Flash HT‐EA or Carlo Erba 1100 Elemental Analyzer interfaced to a Delta V Advantage Isotope‐Ratio Mass Spectrometer (Thermo Finnigan). Laboratory standards of leucine, caffeine (IAEA‐600), and fish protein powder were analyzed in each C and N isotope run. Hydrogen isotope ratios were measured on H_2_ gas derived from high‐temperature (1,030°C) Cr‐based reactor using a Uni‐prep carousel (Eurovector) and elemental analyzer (Thermo Flash HT/EA; Thermo Finnigan) coupled to a continuous‐flow isotope‐ratio mass spectrometer (Delta V Advantage; Thermo Finnigan). Isotope values are reported in parts per thousand (‰) deviations from the international standard: V‐PDB for *δ* ^13^C, AIR for *δ* ^15^N, and VSMOW for *δ* ^2^H. Within‐run standard deviation of laboratory standards replicates was lower than 0.1‰ for *δ* ^13^C and *δ* ^15^N, and 2.0‰ for *δ* ^2^H.

Measurements of nonexchangeable *δ* ^2^H in fish samples were determined by using the comparative equilibration method (Wassenaar & Hobson, 2003). The H isotopic exchange between ambient and sample moisture was controlled in the Uni‐Prep carousel at 60°C (Soto et al., 2017). Samples and standards were loaded into the Uni‐prep carousel and flushed with helium for 30 min using a modified EA configuration that allows a parallel flow of the reference flow from the EA to go through the carousel. The carousel was then evacuated, and 20 μl of water of known isotopic composition was injected to equilibrate samples and standards in similar manner. After equilibration, the carousel was flushed with helium for 2 hr before analysis. Reference materials of similar H exchangeability properties were used in each batch run for isotope data calibration (CBS, −157.0 ± 0.9‰; and KHS, −35.3 ± 1.1‰; Soto et al., 2017). Laboratory fish muscle standard was analyzed as well in each analytical run as a QA/QC, and the *SD* of replicate analyses was ±1.0‰ (*n* = 14). Due to unknown H exchangeability properties of other samples such as invertebrates and primary producers, measurements of nonexchangeable *δ* ^2^H in these samples were determined following a similar procedure as for fish samples, but samples were equilibrated with two waters of contrasting known isotopic composition (−435.5‰ and +945‰). We estimated the proportion of H exchangeability (*f*_ex_) for each sample and the nonexchangeable *δ* ^2^H value (*δ* ^2^H_n_) was calculated using the following equation:

(1) $$\delta^{2}H_{\text{total}}=f_{\text{ex}}*\delta^{2}H_{\text{ex}}+\left(1-f_{\text{ex}}\right)*\delta^{2}H_{n}$$

where *δ* ^2^H_total_ is the measured hydrogen isotopic composition of sample, and *δ* ^2^H_ex_ is the *δ* ^2^H value of exchangeable hydrogen and ultimately the isotope value of the equilibration water used. These procedures that control the exchangeable hydrogen make our *δ* ^2^H values comparable among analytical runs, sampling events, sample treatment, etc.

### 2.5 Mixing models

Prior to mixing models, we calculated the probability of that the isotope values of consumers being inside the proposed mixing polygon using a Monte Carlo simulation method with 1,500 iterations (Smith, Mazumder, Suthers, & Taylor, 2013). Consumers were within the mixing polygon with a probability higher than 95%, except for seven fish and three invertebrate samples (~7%), mainly due to ^13^C‐depleted values, that were excluded from the mixing models. In addition, samples without values for one isotope were not included in the model (one fish and six invertebrate samples).

We performed Bayesian stable isotope mixing models (MixSIAR, Stock & Semmens, 2013) to estimate the relative contributions of primary energy sources: terrestrial vegetation (C_3_ and C_4_ plants) and aquatic algae, incorporated along the Congo River food web components. The presence of aquatic macrophytes in the system that could support the food web was mainly limited to the emergent *Vossia cuspidata* (C_4_ plant), which form meadows in the Congo basin. In our study region, these plants are present in the riparian zones, but in very localized patches, along the main Congo River and in the main Lomami, but more abundant near river confluences or in some of the tributaries such as the Lobaye (tributary of the Lomami). Other sampled aquatic plants (i.e., *Eichhornia crassipes*, *Salvinia natans*, *Nymphaea* sp.) with isotope values characteristic of C_3_ plants (see Results) were found in a very low abundance and were not considered in the model as primary energy source as their collection was anecdotal. We assumed the isotopic composition of algae production to be close to −150 ± 27‰ for *δ* ^2^H based on the river water *δ* ^2^H value (~0‰; Brett, Holtgrieve, & Schindler, 2018) and −30 ± 3‰ for *δ* ^13^C based on isotope values of DIC (dissolved inorganic carbon; see Discussion). This algal production included potential pelagic (phytoplankton) and benthic (periphyton) microalgae together because unfortunately we were not able to obtain a pure sample for any of these items in our sampling. The reported algal discrimination from water or DIC for either phytoplankton and periphyton was reported to be similar for *δ* ^2^H in a meta‐analysis study (Brett et al., 2018), but some differences (~4‰) can be found for *δ* ^13^C (Vander Zanden & Rasmussen, 1999). To accommodate for the additional uncertainty associated with the variability of C isotope fractionation during photosynthesis, the DIC isotopic variation was then increased by 50%. In a first model, consumers were divided in fish and invertebrates, and in a second model, only fish species were included and were divided in feeding groups. Each factor was included as a fixed effect in the models. We applied discrimination factor values of 0.4 ± 1.3‰ for *δ* ^13^C (Post, 2002) and no discrimination for *δ* ^2^H. We conducted two different models, one using *δ* ^13^C data only and another using *δ* ^13^C and *δ* ^2^H data together for comparison purposes. We did not include *δ* ^15^N values into models since *δ* ^15^N values for different primary producers were in the same range and their resolution is limited. The models were run with 300,000 iterations, removing 150,000 for burn‐in and thinning by a factor of 50. These Bayesian approaches are based on Markov chain Mote Carlo (MCMC) methods, and the burn‐in period diminishes the influence of the starting values.

For the inclusion of *δ* ^2^H into the mixing models, we need to account for the total contribution of environmental water into tissue H (*ω*_compound_) to remove the effects of H derived from water for each subsequent trophic level up to the baseline primary source in order to correctly evaluate autochthonous and allochthonous primary sources. We corrected the measured *δ* ^2^H values for aquatic organisms from the Congo River 2012 to take into account this trophic compounding effect as follows (Solomon et al., 2009):

(2) $$\delta^{2}H_{\text{consumer}}=\left(\omega_{\text{compound}}\times \delta^{2}H_{\text{water}}\right)+\left(\left(1-\omega_{\text{compound}}\right)\times \delta^{2}H_{\text{source}}\right)$$

(3) $$\omega_{\text{compound}}=1-{\left(1-\omega \right)}^{\tau }$$

where *δ* ^2^H_consumer_, *δ* ^2^H_water_, and *δ* ^2^H_source_ are the *δ* ^2^H value of consumer, environmental water, and source, respectively, and *τ* is the difference in trophic position between the consumer and the primary source. We estimated *τ* equal to the consumer trophic position (TP) minus 1, where TP = [1 + (*δ* ^15^N_consumer_ − *δ* ^15^N_source_)/3.4], where *δ* ^15^N_consumer_ is the *δ* ^15^N value of the consumer; *δ* ^15^N_source_ is that of the primary sources (+5.6‰). For these calculations, we need to determine or assume some parameters. First, we assumed that organism body water comes principally from environmental water. Second, the proportion of tissue H derived from environmental water (*ω*) was assumed to be constant along the food chain of each consumer and we applied a value of 28% for fish and 40% for invertebrates. These values were obtained from Soto et al. (2013) and using the correction equations associated with the new assigned hydrogen isotope values of reference materials for analytical measurements (Soto et al., 2017).

## 3 RESULTS

**Figure 2:**
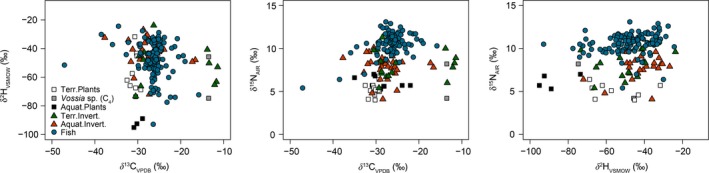
Biplots of the stable isotopic composition of C, N, and H of primary producers and main consumers (invertebrates and fish) from the mainstream Congo River in 2012. All isotope values are raw data without removing the effect of water contribution (H) or without applying any C and N trophic discrimination

We observed a clear difference among food web components from the mainstream Congo River when using combined C, N, and H isotope data. All stable isotope data (*δ* ^13^C, *δ* ^15^N, *δ* ^2^H) from the mainstream Congo River and its tributaries are presented as biplots in Figure 2 (all data from mainstream for which also H isotopes were available) and Figure S1 (*δ* ^13^C and *δ* ^15^N results from the mainstream and tributaries). Values of *δ* ^13^C were much higher in the C_4_ grass *Vossia* sp. (−13.1 ± 1.0‰) relative to the POM (−28.1 ± 1.3‰) and terrestrial C_3_ (−31.3 ± 2.2‰) and aquatic (−29.6 ± 4.0‰) plants (Figure 2, Figure S1). The spread of *δ* ^13^C for the consumers was generally larger in the mainstream Congo than in the tributaries. The ranges of *δ* ^13^C and *δ* ^15^N values for fish spanned from −40.9‰ to −21.2‰ and 9.3‰ to 16.0‰ in Lubilu, from −41.4‰ to −24.6‰ and 6.6‰ to 14.8‰ in Lomami, and from −47.1‰ to −15.8‰ and 5.4‰ to 15.2‰ in the mainstream Congo, respectively, and this isotope variation of these communities clearly overlapped between seasons or sampling periods for each site (Figure S1). In the case of aquatic invertebrates, those ranges of isotope values were from −41.4‰ to −20.9‰ for *δ* ^13^C and 7.4‰ to 16.5‰ for *δ* ^15^N in Lubilu, from −31.0‰ to −19.2‰ for *δ* ^13^C and 3.2‰ to 12.0‰ for *δ* ^15^N in Lomami, and −37.7‰ to −16.6‰ for *δ* ^13^C and 4.1‰ to 10.1‰ for *δ* ^15^N in the mainstream Congo, respectively. For terrestrial invertebrates, isotope values ranged from −32.0‰ to −11.4‰ for *δ* ^13^C and 3.7‰ to 15.2‰ for *δ* ^15^N in Lubilu, Lomami, and the mainstream Congo. Terrestrial invertebrates were separated in two groups, one being enriched in ^13^C (*δ* ^13^C> −17‰) similar to the isotopic composition of *Vossia* sp. (C_4_ plant), which includes adult tabanids (Diptera), grasshoppers (Acrididae), and aquatic gastropods found on *Vossia* leaves. The more ^13^C‐depleted terrestrial invertebrates had similar *δ* ^13^C values as C_3_ plants. Values of *δ* ^15^N were a good indicator of trophic position with three main average levels: (a) the nitrogen isotopic composition of primary producers including aquatic and terrestrial plants and *Vossia* sp. was 5.6 ± 1.0‰; (b) that of aquatic and terrestrial invertebrates was 8.0 ± 1.4‰; and (c) that of fish was 10.7 ± 1.2‰.

**Figure 3:**
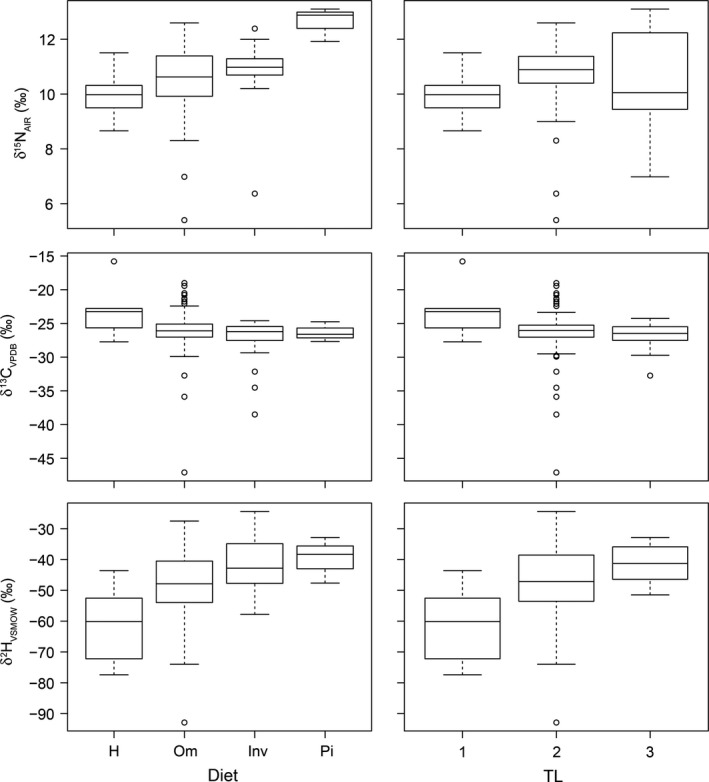
Boxplots of the stable isotopic composition of C, N, and H of fish specimens grouped by dietary habits and trophic level (see Table S1 for details on the fish species involved in each group)

Aquatic plants had lower *δ* ^2^H values (−88 ± 10‰, *n* = 4) than terrestrial plants (−50 ± 14‰, *n* = 8). The range of *δ* ^2^H values for consumers was also large with, for instance, fish having hydrogen isotope values mainly from −80‰ to −20‰ (one value around −90‰). No correlation was found between fish size and *δ* ^2^H values because the standard length of most sampled fish in the mainstream Congo River in 2012 was within a relatively small range between 40 and 150 mm (Figure S3), which avoided any potential effects of size when using *δ* ^2^H (Soto, Hobson, & Wassenaar, 2016). Nonetheless, dietary habits and trophic level had a significant effect on *δ* ^2^H and *δ* ^15^N values (ANOVA, *p* < .01) for fish in the 2012 mainstream sampling (Figure 3). Invertebrate‐feeders had higher *δ* ^2^H values than omnivorous and herbivorous (Tukey's HSD, *p* < .05) and piscivorous higher than herbivorous (Tukey's HSD, *p* = .05). Piscivorous fish had the highest *δ* ^15^N values relative to the other feeding groups (Tukey's HSD, *p* < .05). No significant differences were found among feeding groups for *δ* ^13^C (ANOVA, *p* > .05). Omnivorous fish had the highest variation in all stable isotope data (*δ* ^2^H, *δ* ^13^C, and *δ* ^15^N) indicating their high adaptability of resource use in the system, feeding on plants and animals. Regarding trophic level, the isotopic composition of different fish groups did not differ significantly for *δ* ^13^C and *δ* ^15^N (ANOVA, *p* > .05), but the primary consumers had lower *δ* ^2^H values compared to higher trophic levels (ANOVA, *p* < .01, Tukey's HSD, *p* < .05). These trends indicate that *δ* ^2^H values were influenced by the trophic compounding effect, and confirm that a correction prior their input into the models is needed using the contribution of environmental water (*ω*_compound_).

**Figure 4:**
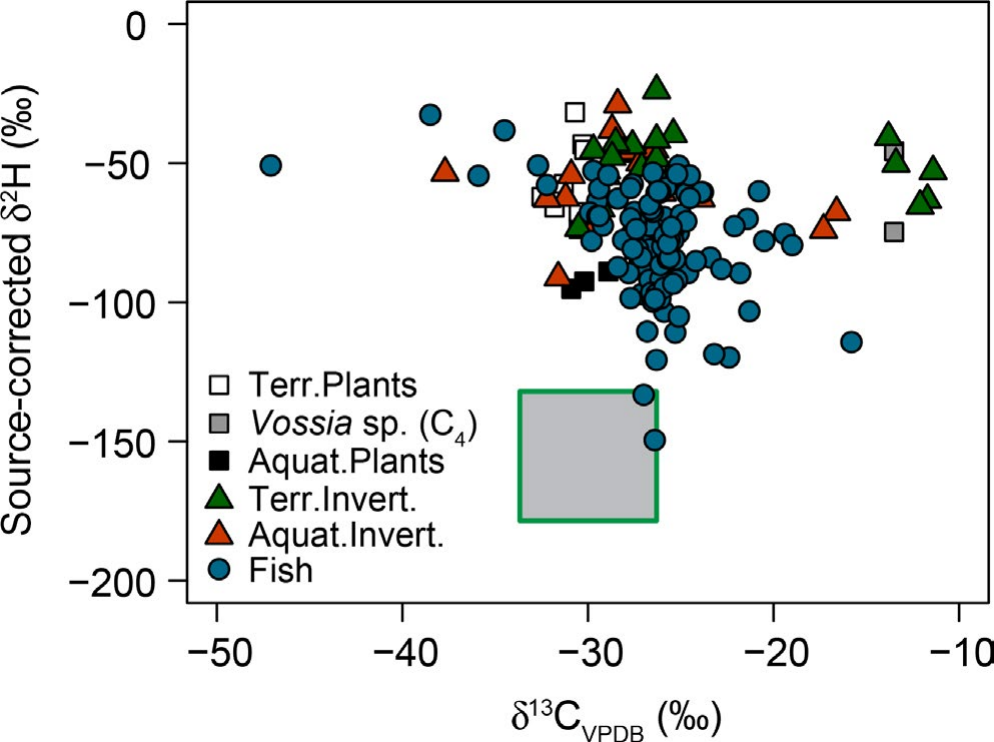
Biplots of the stable isotopic composition of C and H from food web components collected in the mainstream Congo River in 2012, including the expected *δ* ^2^H and *δ* ^13^C values for the aquatic algal production (square), and whose *δ* ^2^H values of aquatic invertebrates and fish were corrected to the primary source (*δ* ^2^H_source_). Carbon isotopic composition of algae was estimated based on the isotopic measurements of DIC samples

**Figure 5:**
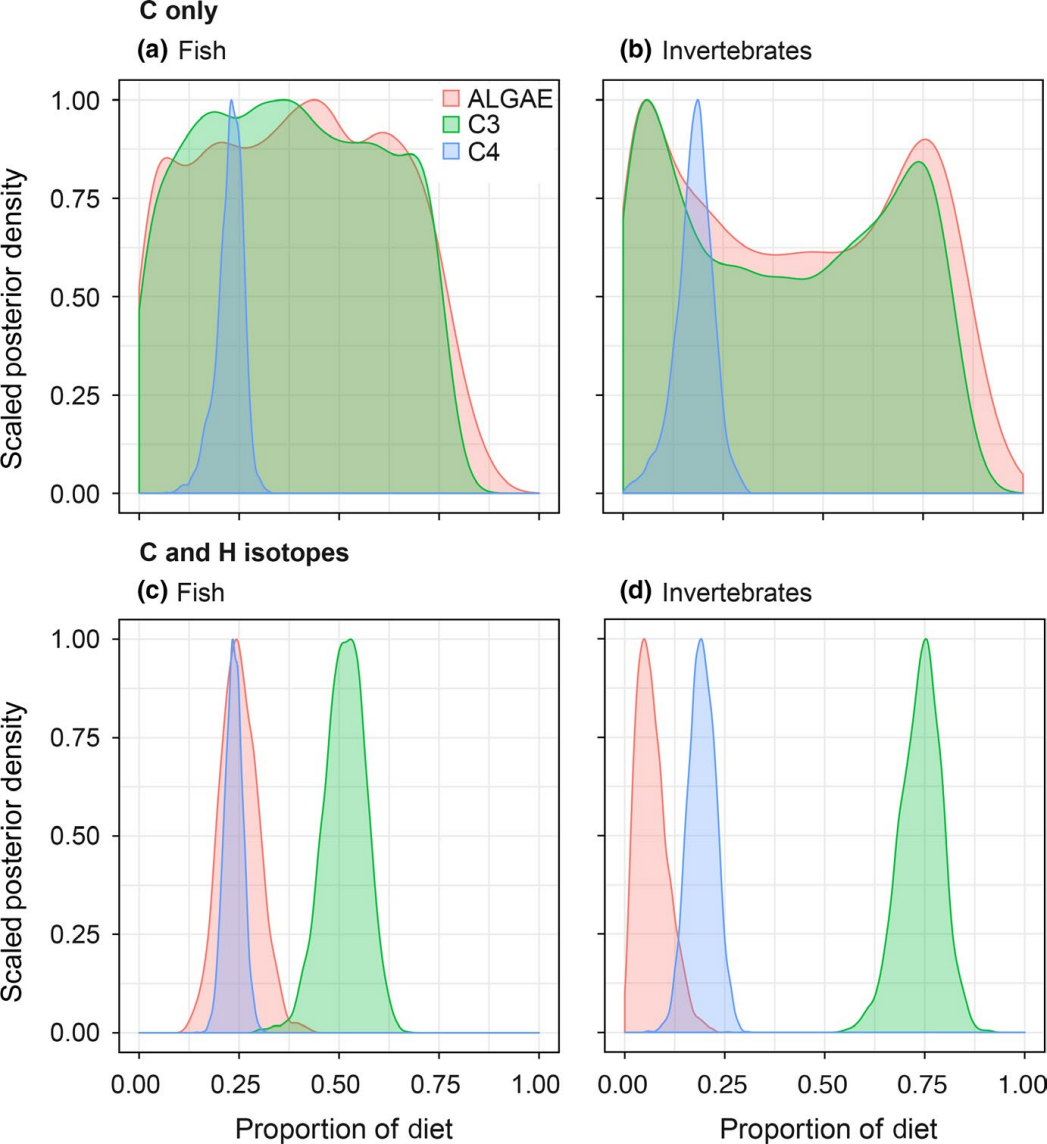
Mixing model results showing the posterior density distributions of proportional contributions of main energy sources (C_3_, C_4_, and phytoplankton) to the Congo River food web components using *δ* ^13^C only and using *δ* ^13^C and *δ* ^2^H data together

Mixing model results revealed a low precision when distinguishing these sources using only C isotopes, with (a) wide posterior distributions of the contributions for C_3_ plants and algae (i.e., *SD* of mean estimate higher than 20% and large 95% credible intervals), and (b) overlapping credible intervals at the level of 95% for all sources and groups assessed (Table 1). Our multiple tracer approach including hydrogen isotopes improved the discrimination between allochthonous and autochthonous sources within the mixing models (Figures 4 and 5). The relative contributions of primary sources incorporated in the fish and invertebrate communities showed a clear predominance of terrestrial C_3_ plants in the consumers (Table 1). Among feeding groups of fish consumers, the main issue with only using C isotopes was similar. We found fish communities to show a higher variation in source‐corrected *δ* ^2^H values than aquatic invertebrates but there were no significant differences in the contribution of autochthonous and allochthonous inputs among different fish feeding groups (Figure 4, Table 1). However, there was a tendency for algae to be a more important primary source for the food chain of herbivorous (mean, 28%) and omnivorous (23%) fish than for invertebrate‐feeders (16%).

**Table 1:** Proportional contributions of primary energy sources incorporated into the Congo River food web components estimated by Bayesian isotope mixing models and using (a) C only and (b) C and H isotope data

	Terrestrial C_3_ plants	Macrophyte C_4_ plants	Algae
1st model—C only			
Fish	37.5 (21.8) [1.4–74.6]	23.0 (3.4) [14.9–28.1]	39.7 (23.0) [1.6–78.9]
Aquatic invertebrates	39.5 (27.0) [0.7–81.6]	18.1 (4.7) [6.6–26.3]	42.3 (28.5) [0.8–87.5]
2nd model—C and H			
Fish	51.5 (5.2) [40.6–60.7]	23.6 (2.2) [19.3–28.0]	24.8 (4.8) [16.1–34.9]
Aquatic invertebrates	74.3 (5.2) [63.0–83.6]	19.2 (3.4) [12.6–25.9]	6.2 (3.9) [1.1–15.9]
1st model—C only			
Herbivores	33.9 (20.3) [2.3–71.4]	26.7 (6.7) [12.9–38.8]	38.5 (22.0) [2.3–78.3]
Omnivorous	34.1 (24.4) [0.9–75.0]	23.0 (4.0) [12.7–28.7]	43.1 (26.2) [1.0–83.4]
Invertebrate‐feeders	35.5 (25.9) [0.9–78.6]	19.8 (4.8) [7.8–26.9]	44.5 (27.9) [1.3–87.6]
Piscivorous	36.9 (27.0) [0.9–84.3]	18.1 (7.7) [3.3–33.2]	44.1 (28.7) [1.2–91.4]
2nd model—C and H			
Herbivores	44.3 (10.4) [23.6–64.1]	27.7 (6.0) [16.4–39.6]	28.1 (9.5) [10.1–46.9]
Omnivorous	53.1 (6.0) [40.5–64.0]	24.0 (2.2) [19.6–28.3]	23.1 (5.8) [12.2–34.9]
Invertebrate‐feeders	62.5 (6.8) [47.9–74.7]	21.3 (3.0) [15.0–26.8]	16.5 (6.3) [4.9–29.4]
Piscivorous	58.3 (13.0) [31.5–82.2]	20.1 (6.9) [6.8–33.6]	21.3 (11.2) [3.2–45.5]

Note

Before inclusion into the model, values of *δ* ^2^H were corrected for the trophic compounding effect (source‐corrected *δ* ^2^H). Median (*SD*) contributions [and 95% credible intervals] are here shown.

In general, the major food item found in the fish stomachs of the fish species collected during our study (Figure S4) was detritus, which obviously includes unknown components of directly ingested detritus material and unidentifiable digested matter, with a different degree of relevance. However, most fish species also contained a considerable amount of terrestrial food items in their stomachs. Among the exceptions are, many insect larvae feeders from the family Mormyridae, and some typical detritus feeders such as *Labeo lineatus* and *Labeo longipinnis*.

## 4 DISCUSSION

The structure of the Congo River food web is composed with three relatively well‐defined trophic levels (primary producers → invertebrates → fish). Our results point toward the significance of terrestrial C_3_ plants (allochthonous sources) for the Congo River food web, but we found a high variability of allochthony in the fish and invertebrate communities of this large tropical aquatic ecosystem. Within the fish communities, a wide range of trophic guilds was present in the Congo River and models including C and H isotopes revealed a tendency of certain variability on source contributions as expected for the fish feeding habits, partly determined by gut content data. Some exceptions of a C_3_‐supported food web included some terrestrial and aquatic invertebrates feeding on *Vossia*‐supported food webs (*δ* ^13^C> −17‰). In general, if we consider both C_3_ and C_4_ plants (terrestrial) as the primary sources, aquatic sources only supported ~20% on fish and ~7% on invertebrates communities. Indeed, it is clear that terrestrial items are important sources for the Congo River food web through either indirect (via ingestion of aquatic invertebrates or other fishes) or direct pathways (consumption of terrestrial animals and plants, see gut content data).

Tropical aquatic ecosystems are often supported to a certain degree by terrestrial inputs (Hoeinghaus, Winemiller, & Agostinho, 2007) although the importance of autochthonous sources in tropical systems such as headwater streams can be relevant (Lau, Leung, & Dudgeon, 2009). In our study, *δ* ^13^C alone could not separate well between aquatic and terrestrial sources in the Congo River ecosystem. The limited power of resolution when using *δ* ^13^C values in some cases has motivated the inclusion of *δ* ^2^H into the isotopic toolbox (reviewed in Vander Zanden, Soto, Bowen, & Hobson, 2016). Our results clearly showed that *δ* ^2^H data complemented the results of the other tracers by separating better the aquatic and terrestrial primary producers (Figures 4 and 5). The source‐corrected *δ* ^2^H values show a high dependency of terrestrial input sources for aquatic invertebrates in 2012 Congo River (Figure 4). When both *δ* ^13^C and *δ* ^2^H data were included, the separation between primary sources in the Congo River mainstream was more efficient and the mixing model estimated a strong contribution of terrestrial C_3_ plants to both aquatic invertebrates and fish (mean, 74% and 52%, respectively; Figure 5). Unfortunately, we did not have *δ* ^2^H measurements for algal material, either as phytoplankton or periphyton, or alternatively any robust primary consumer to serve as a unambiguous algae grazer or planktivore, which only directs us to assume the isotopic composition of algae to be close to −150 ± 27‰ (Brett et al., 2018). Although the empirical values of this source are unknown in the Congo River, we do not expect large changes on our model estimations as demonstrated by the sensitivity analysis performed using contrasting *δ* ^2^H values for the algal source (Table S2), which supports our model assumptions (Brett et al., 2017). A slight change of ~10% on average for the contribution of terrestrial C_3_ plants to diet of the fish community was found when a mean isotopic composition varied between −130‰ and −170‰. Nonetheless, we encourage the sampling of algal material and their consequent correct sample treatment for exchangeable H in future studies using *δ* ^2^H to obtain more accurate model outputs.

Among fish feeding groups, there were slight differences in the algal/terrestrial matter contribution. The higher presence of algal contribution to herbivorous species could be associated to the ingestion of periphyton attached to plants. Alternative causes to this fish *δ* ^2^H variation could be the substantial contribution of aquatic plants into consumer diet (Syväranta, Scharnweber, Brauns, Hilt, & Mehner, 2016) and/or previously described size effects (Soto et al., 2016; Soto, Wassenaar, Hobson, & Catalan, 2011). Both explanations were discarded because firstly the abundance of aquatic plants was very low in the system and secondly the range of fish size classes sampled was also small. Overall, C_3_ terrestrial plants were the dominant energy source for distinct fish groups (~50%) but, in fact, terrestrial C_4_ plants and algal biomass each contributed partly as well and cannot be discarded. Said all this, it is clear that another study based on assessment fish diet could have produced very different results for each feeding group/species, provided that intraspecific variation in feeding ecology of fish communities to be low (Lemmens et al., 2017).

Notably, this study presented a large variation in *δ* ^13^C values of consumers in the Congo River and tributaries, which did not correspond with the isotopic composition of terrestrial plants and POM. This raises the question whether the extended carbon isotopic variability could be explained by a contribution of microalgae and/or the incorporation of methane‐derived biomass.

For the first point, algae *δ* ^13^C values can differ between the tributaries and the mainstream with values covering from ~ −35‰ to −45‰ and −26‰ to −34‰, respectively. These estimations of algae isotope data are based on measured and available *δ* ^13^C‐DIC data from the basin and expected isotopic fractionation during photosynthesis (~ −17‰ to −23‰, Hélie & Hillaire‐Marcel, 2006). Measured values of *δ* ^13^C‐DIC samples during the period of fish sample collection were −23.4 ± 1.7‰ in Lubilu, −20.0 ± 2.1‰ in Lomami, −21.0‰ in the Lobaye, and −12.0 ± 2.0‰ in the mainstream Congo River. In addition, average *δ* ^13^C‐DIC in the Congo River from monitoring samples from 2012 to 2017 collected at Kisangani was −8.8 ± 2.3‰ (*N* = 101; S. Bouillon, unpublished data). If algal production would be a dominant energy source for consumers across all sites, we would thus expect to see a general shift in consumer *δ* ^13^C values, with particularly low *δ* ^13^C in Lubilu, Lobaye, and Lomami, which is not the case (see Figures S1 and S2). Concerning phytoplankton biomass, Descy et al. (2017) showed that there is a higher phytoplankton growth in the main channel of the Congo River during periods of lower discharge and, consequently, lower suspended sediment load—this compared to other tropical systems such as the Amazon and Orinoco rivers. It means that temporal variation in total suspended material loading and light penetration control the phytoplankton biomass of the Congo River, which can support fishes and other organisms from upper trophic levels in periods of low turbidity (Roach, 2013). Estimates of phytoplankton biomass is higher in the main channel (Chl *a*: 1–8 μg/L during low waters) compared to the tributaries (<1 μg/L) where their contribution to the POC pool was minimal (2%–8%, based on high average POC/Chl *a* ratios, ~3,400–3,800 for the mainstream Congo and Lomami, ~5,000 for the Lobaye, and ~9,000 for Lubilu; Descy et al., 2017). Again, in the Congo mainstream, other data such as Secchi depths (cm) and total suspended matter (TSM, mg/L) indicated a high degree of turbidity (31–115 cm and 20–111 mg/L, respectively), particularly relevant in periods of high water. Another potential algal source for the food web could be periphyton. In our study, periphyton biomass was not estimated and we could not separate this from phytoplankton based on empirical and theoretical isotope measurement estimations; thus, we considered this algal package as a whole to be compared with terrestrial C_3_ and macrophyte C_4_ plants in the models. Nonetheless, considering the low Secchi depths (see above), no extensive periphyton growth can be expected except in the littoral zone, for example, attached to *Vossia*. From an energetic point of view, it is unknown if aquatic primary production (on average, in the mainstream, 109 and 229 mg C m^−2^ day^‐1^ during high and falling waters, respectively; Descy et al., 2017) can support fish production, since there are no reliable statistics of either fish biomass or production in the Congo system. The few available estimates of fisheries production indicate that the Congo basin is a highly productive system: potential fisheries production for the rivers of the basin was estimated to be a total of 290,500 t per year (Neiland & Béné, 2008).

Regarding a possible contribution of methane‐derived C, some aquatic invertebrates and a few specimens of demersal fish species (e.g., *Petrocephalus* spp., *Marcusenius* spp., *Pollimyrus* spp.), all of the family Mormyridae or elephant fishes that typically feed on insect larvae, were very depleted in ^13^C (lower than −35‰) relative to the main food web. These values can suggest certain entry of methane‐derived carbon in the ecosystem at a local scale, although the limited number of samples with very ^13^C‐depleted values indicates that was not an important source. As an alternative solution, we conducted an extra mixing model including methane‐oxidizing bacteria as an additional source for the Congo mainstream and including all consumer data. In the absence of direct measurements, the stable isotope composition of methane‐oxidizing bacteria (*δ* ^13^C = −47.5 ± 2; *δ* ^2^H = −80 ± 20) was estimated from measured water *δ* ^2^H and methane *δ* ^13^C (C. Morana and A.V. Borges, unpublished data), and the average expected C and H isotope fractionations for fatty acids in methane‐oxidizing bacteria (Sessions, Jahnke, Schimmelmann, & Hayes, 2002; Whiticar, 1999). While this should be considered only as a very preliminary indication, this alternative mixing model still results in a strong contribution of terrestrial C_3_ plants (median 40% for fish and 71% for invertebrates), a low contribution of algal sources (21% and 4%, respectively), and a relatively low overall contribution of methane oxidizers (9% and 3%, respectively). But again, considering the measured isotope values, this incorporation should be limited to some species that feed in benthic anoxic–oxic interphase environments, and our study is focused on the whole community and assessing specific feeding habits of certain species is out of scope.

Our study provides (a) a unique dataset in one of the most understudied watersheds in the world, (b) a multiple isotope approach including hydrogen isotopes for food web studies, and (c) a progress on the state of art of food web ecology providing new insights into the aquatic ecosystem functioning in tropical habitats. Our results show that we gained power to distinguish sources by using a multiple tracer approach including *δ* ^2^H, *δ* ^13^C, and *δ* ^15^N. While stable isotope techniques have become essential tools to investigate aquatic food webs, the refinement and application of *δ* ^2^H in this context is a very recent development with high potential, but still needs further research and consensus to be applied appropriately and widely as the other tracers. This study is expected to represent a proof of concept for future applications and studies, enhancing our ability to estimate the contribution of allochthonous sources to aquatic ecosystems and thereby supporting the development of effective fisheries management strategies. Given the importance of fisheries to support riparian human populations in these tropical systems, our study points toward the significance of terrestrial sources for an adequate management of aquatic ecosystem and fisheries in the Congo River basin.

## CONFLICT OF INTEREST

None declared.

## AUTHOR CONTRIBUTIONS

DXS, JS, EV, and SB conceived the idea and designed the research. JS, EV, LVW, JB, TM, and SB conducted sample collections and gut content analysis. DXS and SB contributed to stable isotope measurements. DXS analyzed the data. DXS, ED, JS, and SB contributed to the first draft of the manuscript. All authors commented and contributed to the text.

## Data Availability

Stable isotope data used in this study are available at the Dryad Digital Repository: https://doi.org/10.5061/dryad.3n4gt77.
