# Supplementary material for: Terrestrial contributions to Afrotropical aquatic food webs: The Congo River case
Source: Ecol Evol. 2019 Aug 27;9(18):10746–57. doi: 10.1002/ece3.5594 (PMC6787788; doi:10.1002/ece3.5594)
Supplement: Supplementary file 1 [file ECE3-9-10746-s001.docx]

**Supplementary material**

**for**

Terrestrial contributions to Afrotropical aquatic food webs: the Congo River case

David X. Soto, Eva Decru, Jos Snoeks, Erik Verheyen, Lora Van de Walle, Jolien Bamps, Taylor Mambo, and Steven Bouillon

Figure S1. Bi-plots of *δ*^13^C and *δ*^15^N values of primary producers and main consumers (aquatic invertebrates, black triangles; terrestrial invertebrates, white triangles; and fish, grey circles) from the mainstream Congo River and tributaries in 2012, 2013, and 2014.


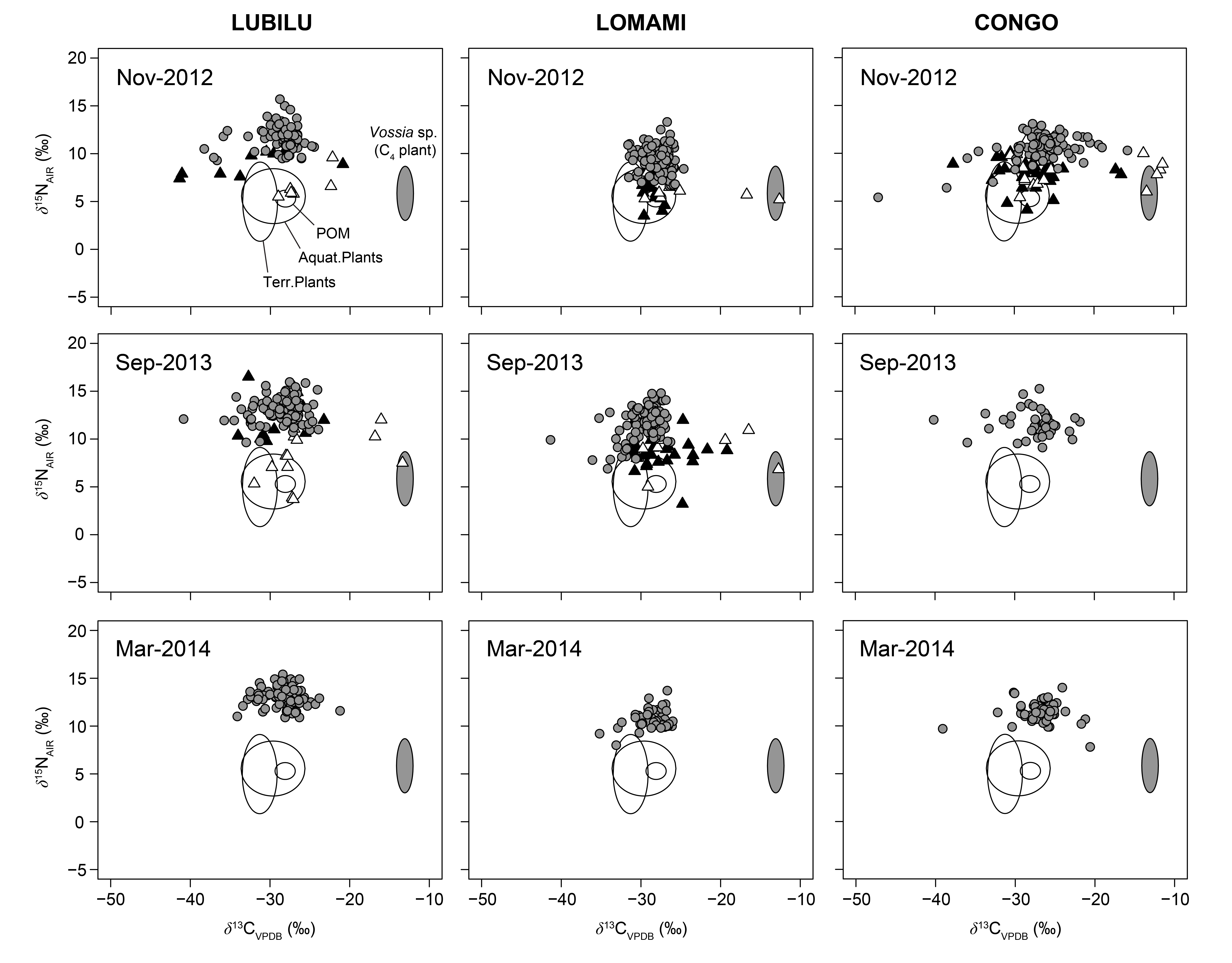


Figure S2. Bi-plots of *δ*^13^C and *δ*^15^N values of primary producers and main consumers (aquatic invertebrates, black triangles; terrestrial invertebrates, white triangles; and fish, grey circles) from Lobeye (tributary of Lomami) collected in 2012, and 2013.


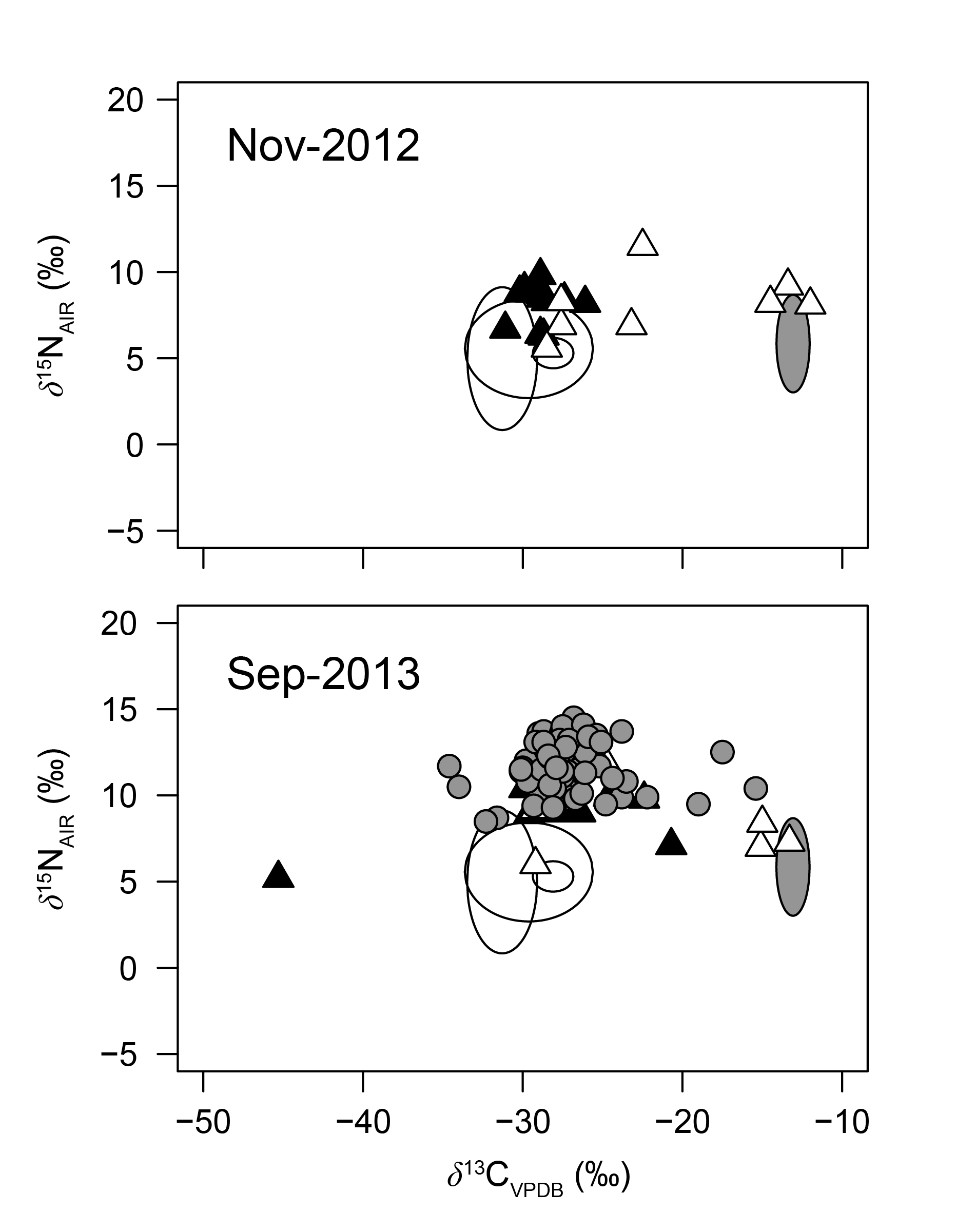


Figure S3. Relationship between size and *δ*^2^H values for fish sampled in the mainstream Congo River in 2012. Colors show the contrasting feeding groups.
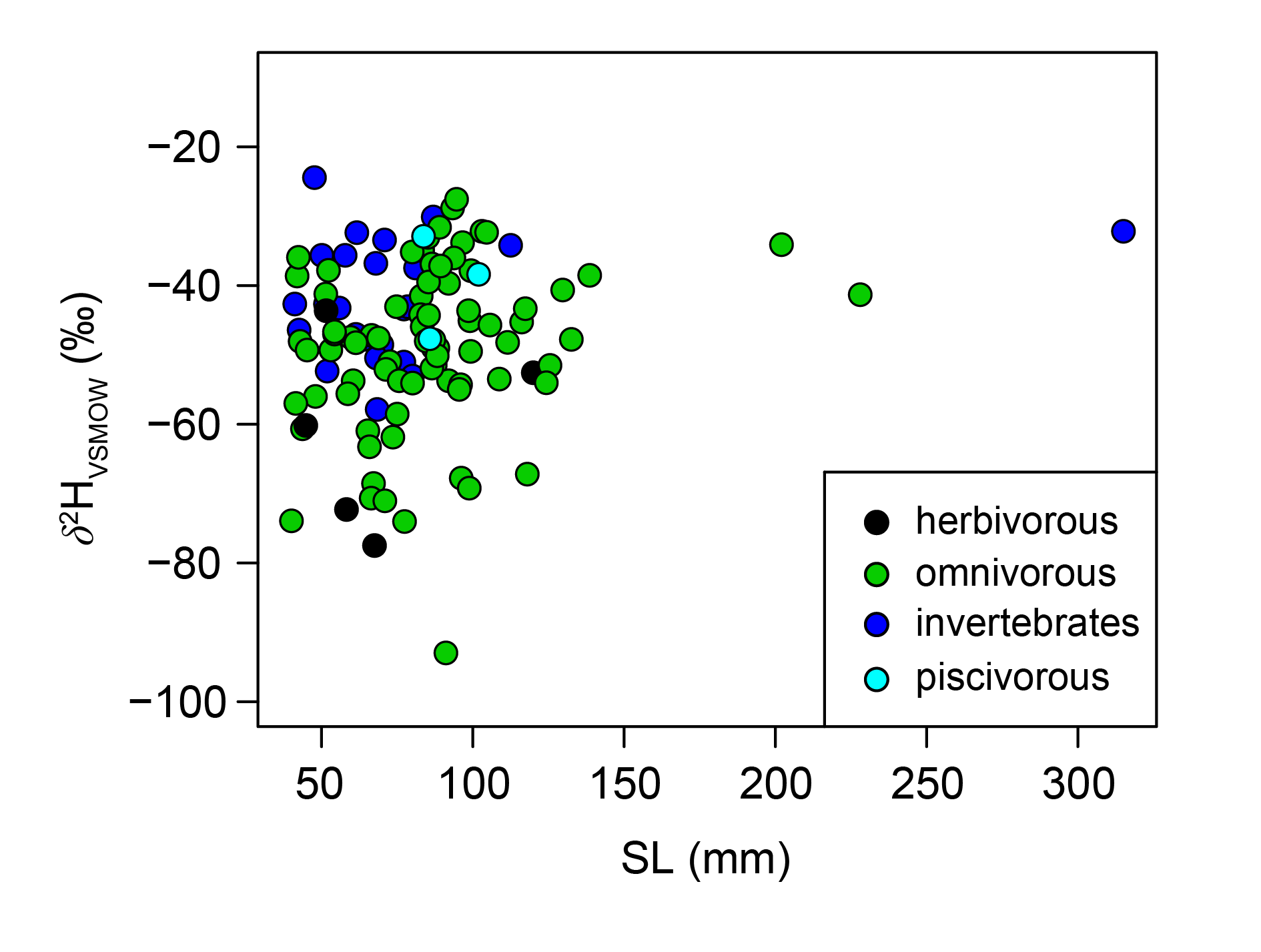


Figure S4. Relative proportions of dry weights of prey items, classified as aquatic animals (dark blue), aquatic plants (light blue), terrestrial animals (brown), terrestrial plants (orange) and all other items, mainly detritus (grey).


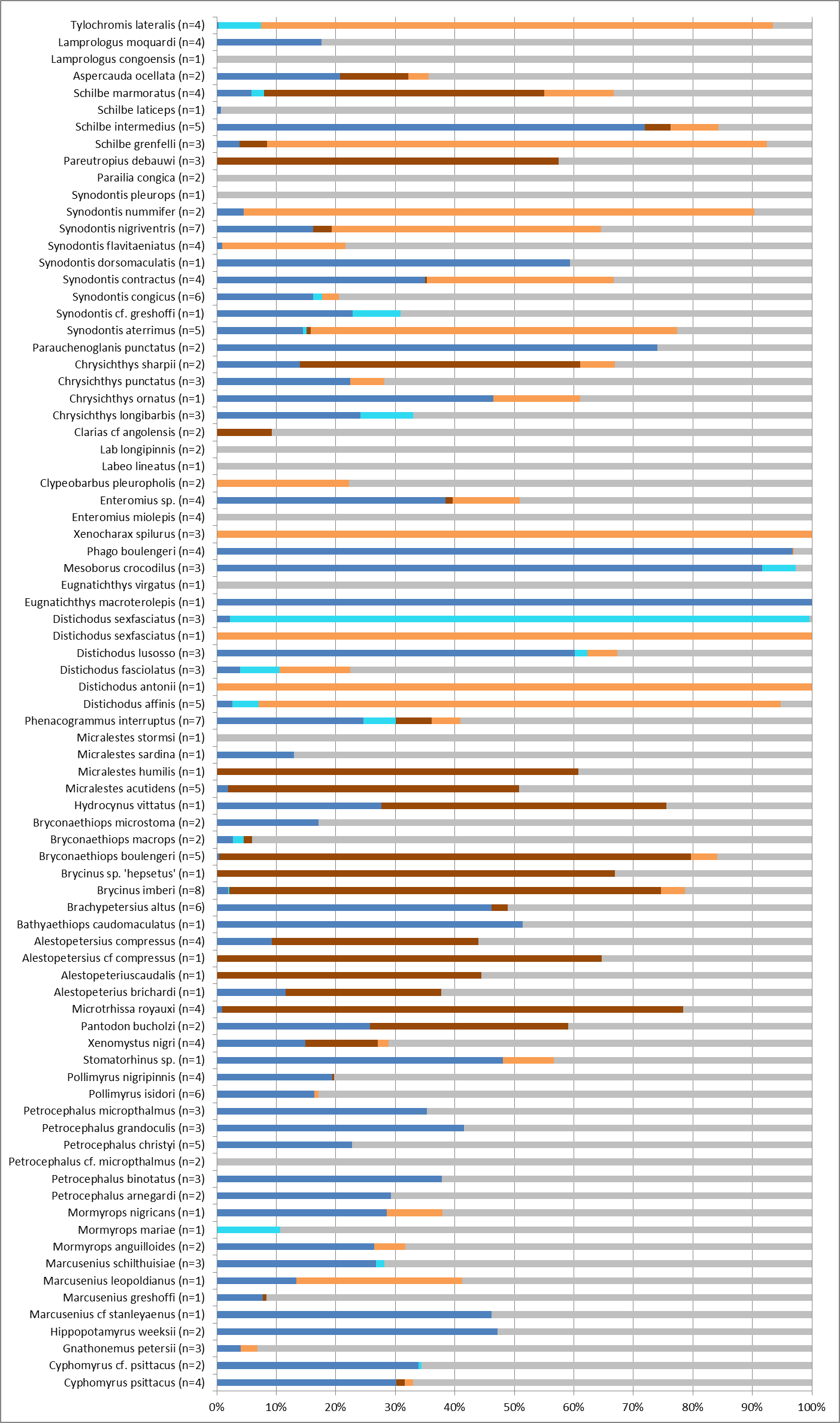


Suppl. Table 1. A summary of fish species collected in the mainstream Congo River during the campaign in November-December 2012. This includes information on dietary habits, trophic level, and sample size analyzed (N). The last column indicates whether dietary habits and trophic levels were obtained from direct gut content data and/or from literature (including citation).

| **Species** | **Diet** | **Trophic level** | **N** | **Reference** |
| --- | --- | --- | --- | --- |
| *Brycinus* "new parvus" | omnivorous | secondary | 1 | Stomach analysis |
| *Brycinus imberi* | omnivorous | secondary | 4 | Stomach analysis; Fishbase |
| *Bryconaethiops boulengeri* | omnivorous | secondary | 5 | Stomach analysis |
| *Bryconaethiops macrops* | omnivorous | secondary | 1 | Stomach analysis |
| *Bryconaethiops microstoma* | omnivorous | secondary | 3 | Stomach analysis; Gosse, 1963 |
| *Chrysichthys congicus* | omnivorous | tertiary | 3 | Extrapolated from congeners |
| *Chrysichthys habereri* | omnivorous | tertiary | 1 | Extrapolated from congeners |
| *Chrysichthys longibarbis* | omnivorous | tertiary | 1 | Stomach analysis |
| *Chrysichthys longipinnis* | omnivorous | tertiary | 2 | Extrapolated from congeners |
| *Chrysichthys sharpii* | omnivorous | tertiary | 1 | Stomach analysis |
| *Clypeobarbus pleuropholis* | omnivorous | secondary | 5 | Stomach analysis |
| *Cyphomyrus psittacus* | omnivorous | secondary | 9 | Stomach analysis |
| *Distichodus affinis* | herbivorous | primary | 1 | Stomach analysis |
| *Distichodus antonii* | herbivorous | primary | 1 | Gosse, 1963; Lévêque et al., 1988 |
| *Distichodus fasciolatus* | omnivorous | secondary | 8 | Stomach analysis |
| *Distichodus lusosso* | omnivorous | secondary | 1 | Stomach analysis |
| *Distichodus sexfasciatus* | herbivorous | primary | 1 | Stomach analysis; Lévêque et al., 1988 |
| *Hydrocynus vittatus* | piscivorous | tertiary | 3 | Stomach analysis; Gosse, 1963; Lévêque et al., 1988; Matthes, 1964 |
| *Labeo longipinnis* | herbivorous | primary | 1 | Lévêque et al., 1988 |
| *Leptocypris modestus* | NA | NA | 1 |  |
| *Malapterurus gossei* | omnivorous | tertiary | 1 | Fishbase |
| *Marcusenius kutuensis* | omnivorous | secondary | 1 | Lévêque et al., 1988; Bernacsek, 1980 |
| *Marcusenius monteiri* | omnivorous | secondary | 1 | Lévêque et al., 1988; Bernacsek, 1980 |
| *Marcusenius schilthuisiae* | omnivorous | secondary | 2 | Stomach analysis |
| *Mastacembelus congicus* | invertebrates | secondary | 1 | Vreven, 2001 |
| *Micralestes humilis* | omnivorous | secondary | 1 | Stomach analysis; Matthes, 1964 |
| *Micralestes lualabae* | omnivorous | secondary | 1 | Jackson, 1961 |
| *Microthrissa congica* | omnivorous | secondary | 5 | Jackson, 1961 |
| *Microthrissa royauxi* | invertebrates | secondary | 6 | Stomach analysis |
| *Paralia congica* | invertebrates | secondary | 2 | Fishbase |
| *Pareutropius debauwi* | invertebrates | secondary | 3 | Stomach analysis |
| *Petrocephalus christyi* | invertebrates | secondary | 5 | Stomach analysis |
| *Petrocephalus micropthalmus* | invertebrates | secondary | 2 | Stomach analysis |
| *Pollimyrus isidori* | invertebrates | secondary | 2 | Stomach analysis |
| *Pollimyrus nigripinnis* | invertebrates | secondary | 3 | Stomach analysis |
| *Pollimyrus plagiostoma* | invertebrates | secondary | 1 | Lévêque et al., 1988 |
| *Protopterus* sp. | omnivorous | NA | 1 |  |
| *Schilbe grenfelli* | herbivorous | primary | 1 | Stomach analysis |
| *Schilbe intermedius* | omnivorous | secondary | 7 | Stomach analysis |
| *Schilbe marmoratus* | invertebrates | secondary | 1 | Stomach analysis |
| *Synodontis alberti* | omnivorous | secondary | 4 | Lévêque et al., 1988 + extrapolation other *Synodontis* spp. |
| *Synodontis angelicus* | omnivorous | secondary | 1 | Lévêque et al., 1988 + extrapolation other *Synodontis* spp. |
| *Synodontis congicus* | omnivorous | secondary | 6 | Stomach analysis |
| *Synodontis greshoffi* | omnivorous | secondary | 4 | Matthes, 1964 |
| *Synodontis pleurops* | omnivorous | secondary | 3 | Lévêque et al., 1988 + extrapolation other *Synodontis* spp. |

NOTE: Stomach content analysis were explored by using two dietary parameters, frequency of occurrence and prey specific abundance. The frequency of occurrence of a specific prey category is the number of stomachs that contain that prey type, as a percentage of all the stomachs that contain prey: [%F_i_=(N_i_/N)*100], with N_i_ being the number of fish with prey *i* in their stomach, and N the number of fish with any prey in their stomach. The prey specific abundance is the weight of the prey category as a percentage of the weight of the total stomach content of all fish (stomachs) that contain that specific prey category: %P_i_=(∑S_i_/∑S_t_)*100, with S_i_ being the stomach content weight of prey *i*, and S_t_ the total stomach content weight of all stomachs that contain prey *i*.

**References Suppl. Table 1**

Bernacsek, G.M., 1980. Introduction to the freshwater fishes of Tanzania. University of Dar-es-Salaam, Department of Zoology, Dar-es-Salaam, Tanzania. 78 p.

Froese, R. and D. Pauly. Editors. 2015. FishBase. World Wide Web electronic publication. www.fishbase.org, version (04/2015).

Gosse, J.P., 1963. Le milieu aquatique et l’écologie des poisons dans la région de Yangambi. Annales du Musée Royal de l'Afrique Centrale, Sciences Zoologiques, 80(116): 113-270.

Jackson, P.B.N., 1961. The fishes of Northern Rhodesia. A check list of indigenous species. The Government Printer, Lusaka. 140 p.

Lévêque, C., M.N. Bruton and G.W. Ssentongo (eds.), 1988. Biologie et écologie des poissons d'eau douce Africains = Biology and ecology of African freshwater fishes. Institut Français de Reserche Scientifique Pour Le Développement en Coopération Collection. Travaux et Documents no. 216.

Matthes, H., 1964. Les poissons du lac Tumba et de la région d'Ikela. Étude systématique et écologique. Ann. Mus. R. Afr. Centr., série in-8°, Sci. Zool. 126:204 p

Vreven, E. 2001. A systematic revision of the African spiny-eels (Mastacembelidae; Synbranchiformes) (Doctoral dissertation, KU Leuven).

Suppl. Table 2. Sensitivity analysis on the influence of the algal isotopic composition in the proportional contributions of primary energy sources incorporated into the Congo River food web components estimated by Bayesian isotope mixing models using C and H isotope data. Before inclusion into the model, values of *δ*^2^H were corrected for the trophic compounding effect (source-corrected *δ*^2^H). Median (SD) contributions [and 95% credible intervals] are shown.

|  | Terrestrial C_3_ plants | Macrophyte C_4_ plants | Algae |
| --- | --- | --- | --- |
| *δ^2^H = ‒130 ± 27 ‰* |  |  |  |
| Fish | 45.1 (6.6) [30.0 – 56.6] | 23.5 (2.4) [18.7 – 28.1] | 31.5 (6.4) [20.4 – 45.6] |
| Aquatic invertebrates | 72.3 (6.2) [58.6 – 82.4] | 19.4 (3.4) [12.4 – 25.8] | 8.0 (5.0) [1.5 – 19.9] |
| *δ^2^H = ‒150 ± 27 ‰* |  |  |  |
| Fish | 51.5 (5.2) [40.6 – 60.7] | 23.6 (2.2) [19.3 – 28.0] | 24.8 (4.8) [16.1 – 34.9] |
| Aquatic invertebrates | 74.3 (5.2) [63.0 – 83.6] | 19.2 (3.4) [12.6 – 25.9] | 6.2 (3.9) [1.1 – 15.9] |
| *δ^2^H = ‒170 ± 27 ‰* |  |  |  |
| Fish | 55.7 (4.2) [46.9 – 63.2] | 23.8 (2.1) [19.7 – 27.7] | 20.6 (3.8) [13.5 – 28.3] |
| Aquatic invertebrates | 75.4 (4.8) [65.2 – 84.4] | 19.2 (3.4) [12.3 – 25.9] | 5.1 (3.1) [0.9 – 13.1] |
|  |  |  |  |
